# Cost‐effectiveness analysis of tumor‐infiltrating lymphocytes biomarkers guiding chemotherapy de‐escalation in early triple‐negative breast cancer

**DOI:** 10.1002/cam4.6656

**Published:** 2023-11-14

**Authors:** Shiqi Li, Yuhan Liu, Peigen Zhang, Mengmeng Wang, Lihua Sun

**Affiliations:** ^1^ Department of Pharmacy Administration, School of Business Administration Shenyang Pharmaceutical University Shenyang China; ^2^ Shanghai Health Development Research Centre (Shanghai Medical Information Centre) Shanghai China; ^3^ The Department of Cardiology General Hospital of Northern Theater Command Shenyang China

**Keywords:** cost‐effectiveness, cytotoxic chemotherapy, early‐stage triple‐negative breast cancer, incremental cost‐effectiveness ratio, quality‐adjusted life years, tumor‐infiltrating lymphocytes

## Abstract

**Background:**

To accelerate the clinical translation of tumor‐infiltrating lymphocytes (TILs) biomarkers for guiding chemotherapy de‐escalation in early‐stage triple‐negative breast cancer (TNBC), cost‐effectiveness evidence is essential but has not been investigated. We intend to evaluate the cost‐effectiveness of using TILs to guiding chemotherapy de‐escalation in patients with early‐stage TNBC from the perspective of the Chinese health service system.

**Methods:**

The hybrid decision‐tree‐Markov model was designed to compare the cost‐effectiveness of cytotoxic chemotherapy guided by whether TILs assay was performed in 50‐year‐old female patients with early‐stage TNBC over a lifetime horizon. In Strategy (1), if TILs testing was performed, patients with TILs values exceeding 30% could be spared from chemotherapy. In Strategy (2), where no TILs testing was performed, all patients were administered chemotherapy following China's clinical practices. Based on the algorithm built by Guyot, the individual patient data were reconstructed from the published Kaplan–Meier curves, and the survival functions were calculated by parametric methods. Cost estimates were valued in Chinese yuan (as per rates in 2022).

**Results:**

In 50‐year‐old female patients with early‐stage TNBC, Strategy (1), which employs TILs testing to guide cytotoxic chemotherapy yielded an additional 0.47 quality‐adjusted life years (QALYs) and saved 40,976 yuan, with an incremental cost‐effectiveness ratio (ICER) of −87,182.98 yuan per QALY gained compared with Strategy (2). This indicates that compared with Strategy (2), Strategy (1) is the dominant scheme. The results were sensitive to utility parameters, discount rates, and treatment costs after relapse. At a willingness‐to‐pay threshold of 85,700 yuan (based on GDP per capita) per QALY, the probability of TILs being cost‐effective was almost 100%.

**Conclusions:**

The application of biomarkers (TILs) to guide decisions for chemotherapy de‐escalation is a cost‐effective strategy for early‐stage TNBC patients and deserves to be widely promoted in clinical practice.

## Graphical abstract

Tumor‐infiltrating lymphocytes (TILs) testing is a cost‐effective strategy for guiding chemotherapy de‐escalation in early‐stage triple‐negative breast cancer (TNBC) patients, according to a study from Chinese health care providers. Using TILs testing can spare patients unnecessary chemotherapy while adding an additional 0.47 quality‐adjusted life years (QALYs) and saving costs. The study found that using TILs testing to guide chemotherapy in early‐stage TNBC patients was almost 100% cost‐effective at a willingness‐to‐pay threshold of 85,700 yuan per QALY. This highlights the importance of incorporating cost‐effectiveness evidence into clinical decisions for biomarker‐guided chemotherapy de‐escalation.

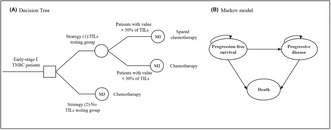

## 1 BACKGROUND

Triple‐negative breast cancer (TNBC), defined by a lack of HER2 (human epidermal growth factor receptor 2), progesterone, and estrogen expression,^1^ accounts for 19% of breast cancer cases.^2^ TNBC has always been a challenging breast cancer subtype to treat. It offers patients the worst prognosis of all breast cancers. Its highly proliferative tumors show an aggressive phenotype and result in high local and distant recurrence.^3^ With limited treatment options for early‐stage TNBC, cytotoxic chemotherapy remains the treatment mainstay.^4^, ^5^ While cytotoxic chemotherapy can increase the chances of survival in patients with early‐stage TNBC, it also has a substantial cost burden and associated toxicities. Cytotoxic chemotherapy may lead to cardiac and myelosuppressive risks, life‐threatening infectious complications, and long‐term peripheral neuropathy, affecting patient function and quality of life.^6^, ^7^, ^8^ Thus, judicious decisions regarding risks and benefits should be made before administering cytotoxic chemotherapy. Several clinical and biological factors indicate that not all early‐stage TNBC patients gain the same degree of benefit from cytotoxic chemotherapy.^9^, ^10^, ^11^ A subset of patients with early TNBC have a good prognosis with a less than 10% risk of 5‐year distant recurrence of their cancer.^10^, ^12^, ^13^, ^14^ In these patients, cytotoxic chemotherapy does not offer significant benefits and may lead to additional adverse effects and costs. Thus, omission or de‐escalation of chemotherapy may be considered if patients at low risk of recurrence can be correctly identified.

Treatment de‐escalation in patients with TNBC has been challenging because of limited treatment options other than cytotoxic chemotherapy and the limited use of prognostic biomarkers outside the pathology context.^10^ In recent years, research has shown that tumor‐infiltrating lymphocytes (TILs) are a robust and independent prognostic factor for early‐stage TNBC, providing important prognostic information for estimating survival.^15^, ^16^, ^17^, ^18^ Studies have shown that Stage‐1 TNBC patients with TIL assay values exceeding 30% have excellent survival outcomes without adjuvant chemotherapy. This suggests that a subset of patients could be spared adjuvant chemotherapy, as the expected survival benefit on the absolute risk scale may not outweigh the associated morbidity.^19^, ^20^, ^21^ Most researchers agree that TILs are the first prognostic biomarkers of early‐stage TNBC, identifying a cutoff value of 30% as appropriate for potential chemotherapy de‐escalation.^20^, ^22^ That is, TILs can help doctors identify early‐stage TNBC patients who can safely spare chemotherapy. This will reduce the toxicity and cost of chemotherapy without sacrificing the survival outcomes. The TIL Working Group has developed a method to quantify TILs, which has improved their reproducibility and assay validity. At present, the TILs biomarker has been incorporated into many international guidelines for early‐stage breast cancer, including the 2019 St Gallen consensus conference,^23^ the European Society of Medical Oncology (ESMO) Guidelines,^24^ and the World Health Organization (WHO) Blue Book on breast tumor classification.^25^ This biomarker has reached level‐IB‐evidence.

Although the clinical utility and assay validity of TILs to guide chemotherapy in patients with early‐stage TNBC have been demonstrated, the cost‐effectiveness is still unknown. However, it is significant and requisite for policymakers and clinical decision‐makers to establish this cost‐effectiveness, particularly for developing countries like China. At this point, there is no evidence of TILs' cost‐effectiveness in China or worldwide. Thus, we intended to assess the cost‐effectiveness of TILs assays used for chemotherapy de‐escalation in patients diagnosed with early‐stage TNBC to contribute to the current recommendations regarding its use.

## 2 METHODS

This study considered the Chinese context for its cost‐effectiveness analysis of TILs. The study followed the recommendations of the Consolidated Health Economic Evaluation Reporting Standards (CHEERS).^26^

### 2.1 Model description and assumptions

The hybrid decision‐tree‐Markov model was designed to project the expected clinical outcomes and treatment costs over the lifetimes of 50‐year‐old female patients with early‐stage TNBC. These projections consider two different strategies from the perspective of the Chinese health service system. A decision tree allocated a patient cohort to one of the two strategies, in which patients either received or did not receive TILs testing. In Strategy (1), the patient's TILs value was used to guide the de‐escalation of treatment in female patients with early‐stage TNBC. Patients with TILs values greater than 30% could be spared chemotherapy and entered Markov Model 1 (M1). Patients with TILs less than 30% were administered adjuvant chemotherapy according to the AC‐T regimen of anthracyclines (A), including epirubicin, pirarubicin, and doxorubicin; Taxus (T), including taxel and docetaxel; and Cyclophosphamide (C). After this chemotherapy regimen was administered, patients were entered into Markov Model 2 (M2). In Strategy (2), patients with early‐stage TNBC were not tested for TILs. All patients in Strategy (2) received chemotherapy according to the current treatment standard, which was the TC regimen of Taxus (T), including taxel and docetaxel; and Cyclophosphamide (C). After chemotherapy was administered, patients were entered into Markov Model 3 (M3).

**FIGURE 1:**
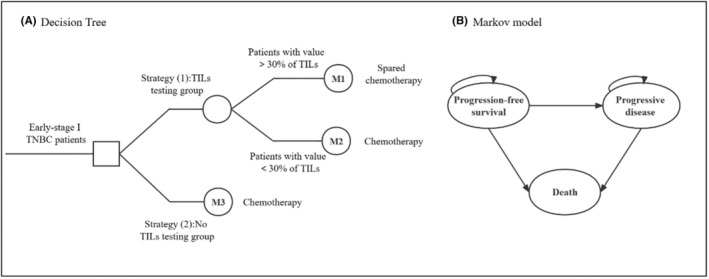
The hybrid decision tree‐Markov model. A Schematic of decision tree. B Markov states and transitions.

Three mutually exclusive Markov states were defined as follows: progression‐free survival (PFS) (often referred to as disease‐free survival [DFS]^27^), progressive disease (PD), and death. All patients entering the Markov model were simulated from the PFS state, and depending on the corresponding transition probabilities, the patients would either remain in the PFS state for the next cycle or move to the relapse or death states. Patients who progressed to the relapse state could not return to the PFS state. It was only possible for these patients to remain in the PFS state or enter the death state. The death state was the state of absorption, and entering this state ended the simulation. This study is not an economic evaluation of breast cancer treatment options, but rather focuses on long‐term survival of breast cancer. Therefore, we did not choose the traditional cycle length of chemotherapy such as 21 or 28 days. A cycle length of 1 year was chosen because most published transition probabilities post‐recurrence was presented as annual rates and a year represents the appropriate time‐frame for follow‐up of women who remain disease free. Therefore, the model was constructed using a 1‐year cycle length and a lifetime horizon. The lifetime horizon was defined as the remaining lifetime of patients using the average maximum life expectancy in China of 77 years. Figure 1 shows the hybrid decision‐tree‐Markov model.

In this research model, the selection of treatment regimen was based on the “Chinese Society of Clinical Oncology (CSCO) Breast Cancer Diagnosis and Treatment Guidelines 2022 edition”. In the TILs‐testing group, TILs values below 30% indicate a relatively higher risk of recurrence. As per the CSCO guideline, І‐level recommends AC‐T chemotherapy regimens in such cases. Conversely, in the no‐TILs‐testing group, for newly diagnosed early‐stage TNBC patients, AC or TC regimens are commonly employed. However, the cost of the TC regimen is higher than that of the AC regimen. In developing countries with unbalanced health care systems, such as China, hospitals generally lean toward the more cost‐effective AC regimen. If no progression is observed after chemotherapy, further medication will not be administered until progression occurs. After progression (recurrence), second‐line treatment will be initiated. The proportion of patients entering the progression status is calculated based on the survival curve and metastasis probability. Second‐line treatment options include radiotherapy, chemotherapy, targeted therapy, immunotherapy, or surgery, which will be determined by the doctor.

To prevent complex confounding factors from complicating the research question, we may need to make appropriate assumptions in the model. In this study, the following assumptions were made: we did not consider recommendations of II‐level or III‐level in the CSCO guideline for TAC or FEC‐T regimens for patients with BRCA mutations, nor did we consider treatment scenarios involving the administration of capecitabine rescue therapy after failure of TAC chemotherapy.

### 2.2 Population

All the simulated patients were female. Given that the average age of TNBC diagnosis in China is approximately 45–55 years,^28^ patient simulations were carried out from the starting age of 50 years in a yearly cycle. The average patient weight and height were 59 kg and 158 cm, respectively. These values were reported by the Nutrition and Chronic Diseases of Chinese Residents in 2020.^29^ The average body surface area was calculated as follows: average body surface area (m^2^) = 0.0061 × height (cm) + 0.0128 × weight (kg) − 0.1529.^29^ The model starting cohort was assumed to be 10,000 people.

### 2.3 Clinical data and probabilistic parameters

#### 2.3.1 Survival probabilities and extrapolations

In cost‐effectiveness studies, when the Markov simulation time ranges are much longer than that of randomized controlled trials (RCTs), there is a general preference to use the fitted parameter distribution rather than applying the original data from RCTs. Loi et al.^19^, ^20^ reported the respective Kaplan–Meier survival curves of overall survival (OS) and disease‐free survival (DFS) in early‐stage TNBC patients with TILs greater than 30% who were spared adjuvant chemotherapy and in patients with TILs less than 30% who underwent chemotherapy. Corresponding to the Markov model, we refer to these as M1‐DFS, M1‐OS, M2‐DFS, and M2‐OS, respectively. Jones et al.^30^ reported OS and DFS curves in patients undergoing chemotherapy with TC regimens. Similarly, these models are referred to as M3‐DFS and M3‐OS. Based on these curves, individual patient data (IPD) were reconstructed using the algorithm built by Guyot et al.^31^ Then, the parametric method was used to calculate survival functions. Parametric log‐logistic, exponential, Weibull, Gompertz, log‐normal, and generalized Gamma models were fitted to patient‐level survival time data to estimate the rates of the observed 5‐year follow‐up period, which were then extrapolated to the lifetime years. Corresponding with guidelines for parametric survival analysis for health‐economic applications,^32^ the best parameter distributions were selected by the Akaike information criterion (AIC), Bayesian information criterion (BIC), visual comparison of the model with Kaplan–Meier survival curves, and the best fit from simulation results. Analyses were conducted using Stata version 15.1. and the GetData Graph Digitizer 2.26. The optimal parameters for each model are presented in Table 1. Taking the parametric Weibull model as an example, the time‐dependent transition probability is denoted by:

$$\mathrm{tp}\left(t\right)=1-\exp\left\{\lambda {\left(t-u\right)}^{\gamma }-\lambda t^{\gamma }\right\}$$

where the *λ* parameter defines the scale of the distribution, and the (ancillary) γ parameter defines the shape. The length of the Markov cycle is defined as u, and t represents time.

**TABLE 1:** Parameters of the survival curves.

Curves type	Optimal fitting model	λ	γ
M1‐DFS	Log‐normal	2.880	0.061
M1‐OS	Log‐normal	3.240	−0.120
M2‐DFS	Weibull	0.002	1.040
M2‐OS	Log‐normal	5.920	0.360
M3‐DFS	Exponential	0.034	–
M3‐OS	Weibull	0.015	1.160

*Note*: M1‐DFS is the DFS curve for Markov Model 1; M1‐OS is the OS curve for Markov Model 1, and so on.

#### 2.3.2 Other probabilities

This model assumed that transition probabilities from the PFS state to the death state were the average age‐specific death rate for women in 2022 in China.^33^ This assumption was applied to both strategies. In addition, approximately one‐third of the patients had at least 30% TILs in Strategy (1).^19^, ^20^

### 2.4 Cost estimates

The cost was calculated from the perspective of the Chinese health service system and based on the clinical practices of China. Therefore, only direct medical costs were considered, and direct non‐medical costs (e.g., transportation costs) and indirect costs (e.g., loss of productivity) were excluded. The cost of the TILs testing was provided by Wisee Biotechnology Inc., which quoted a price of 3000 yuan per person tested. Direct costs included those costs associated with chemotherapy treatments, recurrence treatments, and patient follow‐up. The average costs of recurrent treatment and follow‐up were based on a cost‐effectiveness analysis of adjuvant therapy for operable breast cancer from a Chinese perspective.^34^ All cost data are adjusted for 2022 Price levels using China's Consumer Price Index (CPI). Detailed cost data are shown in Table 2. The chemotherapy treatments included the following:

1. Chemotherapy drugs: First, we considered the AC‐T regimen for four cycles for patients with TILs less than 30%: Epirubicin (10 mg vial), 100 mg/m^2^ for 16 vials per cycle; cyclophosphamide (200 mg vial), 600 mg/m^2^ for 5 vials per cycle; docetaxel (20 mg vial), 80 mg/m^2^ for 7 vials per cycle). Second, we considered the TC regimen for four cycles for patients with early‐stage TNBC who were not tested for TILs: Docetaxel (20 mg vial), 75 mg/m^2^ for 6 vials per cycle, epirubicin (10 mg vial), 100 mg/m^2^ for 5 vials per cycle).
Prophylaxis drugs (Concomitant medication): The AC‐T regimen had a high risk of emesis and a moderate risk of myelosuppression. To prevent vomiting, patients received Palonosetron (1 vial per cycle), aprepitant (1 vial per cycle); and dexamethasone (2 vials per cycle). To counter the risk of myelosuppression, patients received granulocyte colony‐stimulating factor (G‐CSF). The TC regimen prevented vomiting with Palonosetron (1 vial per cycle) and dexamethasone (2 vials per cycle). The moderate risk of myelosuppression was also addressed with G‐CSF. The costs (in Chinese yuan) of chemotherapy drugs and prophylaxis drugs were obtained from the 2022 winning bid prices in Chinese provinces.
Managing severe adverse events: Our study only considered Grade 3 and 4 adverse events, and treatment costs and probability of occurrence for these adverse events were derived from a previous report from China.^35^
Chemotherapy administration (including nursing, injection, injection materials, and others) was derived from a pharmacoeconomic evaluation conducted in China.^35^
Testing costs: Testing costs refers to the cost of all relevant examinations and laboratory tests (including routine blood, liver and kidney function, electrocardiogram, etc.) for patients before and after medication, which was derived from a drug economics analysis in China.^36^

**TABLE 2:** Parameters input in the model and their ranges used in the sensitivity analyses.

Type	Base‐case values	Range			Distribution^2^	Source
		Lower	Upper	Rule		
Cost of chemotherapy drugs						
Docetaxel 20 mg vial	139.28	22.6	980	Range^1^	Gamma	Drug centralized procurement
Epirubicin 10 mg vial	79.15	70.1	170	Range^1^	Gamma	Drug centralized procurement
Cyclophosphamide 200 mg vial	23.98	23.95	24.51	Range^1^	Gamma	Drug centralized procurement
Cost of prophylaxis drugs						
Palonosetron 0.25 mg vial	53.8	4.86	220	Range^1^	Gamma	Drug centralized procurement
Oral aprepitan	450	150	574.33	Range^1^	Gamma	Drug centralized procurement
Dexamethasone 5 mg vial	0.58	0.03	39	Range^1^	Gamma	Drug centralized procurement
G‐CSF 100 μg vial	58.98	15.29	73.93	Range^1^	Gamma	Drug centralized procurement
Cost of Managing severe adverse events						
AC‐T regimen	21,175	16,940	25,410	±20%	Gamma	Xu Qiaoping et al.^35^
TC regimen	9201	7360.8	11041.2	±20%	Gamma	Xu Qiaoping et al.^35^
Cost of chemotherapy administration						
AC‐T regimen	3200	2560	3840	±20%	Gamma	Xu Qiaoping et al.^35^
TC regimen	2400	1920	2880	±20%	Gamma	Xu Qiaoping et al.^35^
Testing costs						
AC‐T regimen	1002	801.6	1202.4	±20%	Gamma	Xu Sumei et al.^36^
TC regimen	1005.04	804.03	1206.05	±20%	Gamma	Xu Sumei et al.^36^
Cost of TILs testing	3000	2400	3600	±20%	Gamma	Wisee biotechnology Inc
Cost of recurrence treatments	70319.9	20595.7	120044.2	Literature	Normal	Liubao et al.,^34^ medical institutions database
Cost of follow‐up						
Year 1	1846	–	–	–	–	Liubao et al.,^34^ medical institutions database
Year 2	2128	–	–	–	–	Liubao et al.,^34^ medical institutions database
Year 2+	1564	–	–	–	–	Liubao et al.,^34^ medical institutions database
Utility parameter						
PFS in patients with chemotherapy	0.8	0.73	0.87	Literature	Beta	Li JB et al. 2022,^27^ Earle et al.^37^
PD in patients with chemotherapy	0.5	0.4	0.6	±20%	Beta	Li JB et al. 2022,^27^ Earle et al.^37^
PFS in patients spared chemotherapy	0.94	0.75	1	±20%	Beta	Li JB et al. 2022,^27^ Earle et al.^37^
PD in patients spared chemotherapy	0.73	0.66	0.8	Literature	Beta	Li JB et al. 2022,^27^ Earle et al.^37^
Discount rate %	5	0	10	–	–	–

*Note*: 1 The range of cost was set as the lowest and highest unit price from the Chinese Drug Bidding Database in 2020; 2 The distributions were applied in the probabilistic sensitivity analysis.

Abbreviations: G‐CSF, prevent granulocyte colony stimulating factor; PD, progressive disease; PFS, progression‐free survival.

The Details for all cost calculations of the chemotherapy treatments mentioned above can be found in Table 3.

**TABLE 3:** Details for all cost calculations.

Type	Specification	Dose per unit area	Average body surface area	Required dose (mg)	Time and period	Quantity of drug required per cycle (n)	Price per cycle (Yuan)	Single cycle price (Yuan)	Total price of four cycles
TC regimen									
Docetaxel inj	1 mL:20 mg	75 mg/m^2^	1.5661	117.4575	1/21d*4	6	139.28	835.68	3342.72
Cyclophosphamide inj	200 mg	600 mg/m^2^	1.5661	939.66	1/21d*4	5	23.98	119.9	479.6
Palonosetron hydrochloride inj	5 mL:0.25 mg	–	–	0.25	1/21d*4	1	53.8	53.8	215.2
Dexamethasone sodium phosphate inj	1 mL:5 mg	–	–	10	1/21d*4	2	0.58	1.16	4.64
G‐CSF inj	100 μg	–	–	0.1	1/21d*4	1	58.98	58.98	235.92
Managing severe adverse events	–	–	–	–	–	–	9201	9201	36,804
Chemotherapy administration	–	–	–	–	–	–	2400	2400	9600
Testing costs	–	–	–	–	–	–	1005.04	1005.04	4020.16
Total cots	–	–	–	–	–	–	–	–	54702.24
AC‐T regimen									
Epirubicin hydrochloride inj	10 mg	100 mg/m^2^	1.5661	156.61	1/21d*4	16	79.15	1266.4	5065.6
Docetaxel inj	1 mL:20 mg	80 mg/m^2^	1.5661	125.288	1/21d*4	7	139.28	974.96	3899.84
Cyclophosphamide inj	200 mg	600 mg/m^2^	1.5661	939.66	1/21d*4	5	23.98	119.9	479.6
Palonosetron hydrochloride inj	5 mL:0.25 mg	–	–	0.25	1/21d*4	1	53.8	53.8	215.2
Aprepitan capsules	1*125 mg + 2*80 mg	–	–	–	1/21d*4	1	450	450	1800
Dexamethasone sodium phosphate inj	1 mL:5 mg	–	–	10	1/21d*4	2	0.58	1.16	4.64
G‐CSF inj	100 μg	–	–	0.1	1/21d*4	1	58.98	58.98	235.92
Managing severe adverse events	–	–	–	–	–	–	21,175	21,175	84,700
Chemotherapy administration	–	–	–	–	–	–	3200	3200	12,800
Testing costs	–	–	–	–	–	–	1002	1002	4008
Total cots	–	–	–	–	–	–	–	–	113208.8

*Note*: G‐CSF, granulocyte colony stimulating factor; inj, injection; The costs (in Chinese yuan) of chemotherapy drugs and prophylaxis drugs were obtained from the 2022 winning bid prices in Chinese provinces. Cost estimates were valued in Chinese yuan (as per rates in 2022).

### 2.5 Utility parameter

The effectiveness of treatment was assessed regarding quality‐adjusted life‐years (QALYs), which were calculated by multiplying the length of survival in a given state by the utility of that state. Based on relevant research,^27^, ^37^ utility values in PFS, PD, and death states in the Markov model were assumed to be 0.8, 0.5, and 0, respectively, for patients undergoing chemotherapy. For patients who were spared chemotherapy, these values were assumed to be 0.94, 0.73, and 0, respectively. This is because patients spared from chemotherapy in Strategy (1) do not experience the painful adverse effects and overwhelming financial burden associated with it. Therefore, in theory, their utility values are higher than patients who undergo chemotherapy.

### 2.6 Data analysis

The primary outcome of the study was incremental cost‐effectiveness ratios (ICERs). The formula for this outcome was as follows:

$$\text{ICER}=\frac{\Delta C}{\Delta E}=\frac{C_{1}-C_{0}}{E_{1}-E_{0}}$$

where the ΔC represents the incremental costs, and the ΔE represents the incremental QALYs. $C_{1}$ and $C_{0}$ represent the respective costs of the two strategies, and $E_{1}$ and $E_{0}$ represent the respective health outputs (QALYs) of the two strategies.

The second outcome was the cost‐effectiveness ratio (CER), calculated by dividing treatment costs by QALYs. Cost estimates were valued in Chinese yuan (as per rates in 2022). Costs and QALYs were discounted at the rate of 5% per annum according to the China Pharmaceutical Economics Guide. In this study, we followed international good practice guidelines^38^ for decision‐analytic modeling. The model was programmed and analyzed in Microsoft Excel 2019.

We applied one‐way and probabilistic sensitivity analyses (PSA) to test the robustness of the model. One‐way sensitivity analysis was conducted according to reasonable range adjustments of cost, utility, discount, and other parameters to identify the variables that have a significant impact on the simulation results. A tornado diagram was constructed based on the degree of impact. The price ranges of chemotherapy and prophylaxis drugs were set as the lowest and highest unit prices from the Chinese Drug Bidding Database in 2022. Other parameters were varied by ±20%. A Monte Carlo simulation with 10,000 iterations was performed for PSA. A Monte Carlo simulation scatterplot and acceptability curves were then presented to illustrate PSA results.

### 2.7 Model validation

The cost‐effectiveness model was validated using the Assessment of the Validation Status of Health Economic decision models tool^39^ and evaluated by two external experts (Yuhan Liu and Mengmeng Wang).

## 3 RESULTS

### 3.1 Base‐case analysis

In Strategy (1) (the TILs‐testing group), approximately one‐third of patients were spared chemotherapy and entered the M1 pathway, and approximately two‐thirds of patients entered the M2 pathway after chemotherapy. In Strategy (2) (the no‐TILs‐testing group), all patients received chemotherapy and entered the M3 pathway. The CER for patients in the TILs testing group was 15,445.14; the CER for patients in the no‐TILs testing group was 20675.91. Compared with the additional 9.24 QALYs for no‐TILs testing group, the TILs testing group yielded an additional 9.71 QALYs. The total cost was 150,040 yuan in the TILs testing group and 191,016 yuan in no‐TILs testing group. In summary, the TILs testing group received an additional 0.47 QALY and saved 40,976 yuan. The ICER for the TILs testing group was −87182.98 yuan per QALY gained, suggesting that Strategy (1) (the TILs‐testing group) is the dominant scheme when compared with Strategy (2) (the no‐TILs‐testing group). The calculation details of QALY and ICER are shown in Table 4.

**TABLE 4:** Results of base‐case analysis.

Strategy	Content	Cost (Yuan)	QALYs	Total cost/10000 (Yuan)	Total QALYs/10000	ΔC	ΔE	ICER
TILs‐testing group	M1 pathway	441,758 673.80	36237.89	**150040**	**9.71**	−40,976	0.47	−87182.98
	M2 pathway	663,939,329	60905.77					
	Chemotherapy	364,699,834	–					
	TILs	30,000,000	–					
No‐TILs‐testing group	M3 pathway	778072525.40	92385.78	**191016**	**9.24**			
	Chemotherapy	1,132,088,000	–					

*Note*: M1 pathway is Markov Model 1 pathway, and so on. ICER, incremental cost‐effectiveness ratios. ΔC represents the incremental costs, and the ΔE represents the incremental QALYs.

### 3.2 Sensitivity analyses

**FIGURE 2:**
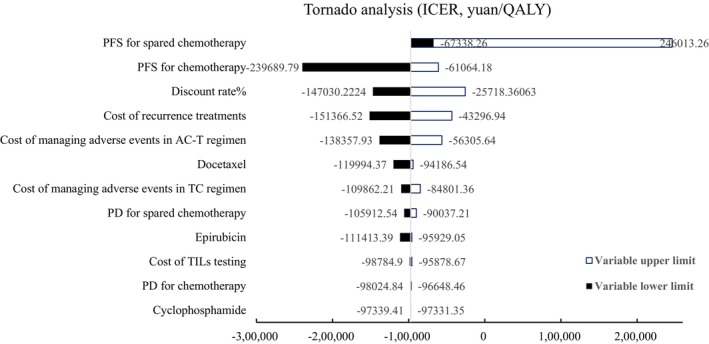
The results of one‐way sensitivity analysis. ICER, incremental cost‐effectiveness ratio; PD, progressive disease; PFS, progression free survival; QALY, quality‐adjusted life year.

**FIGURE 3:**
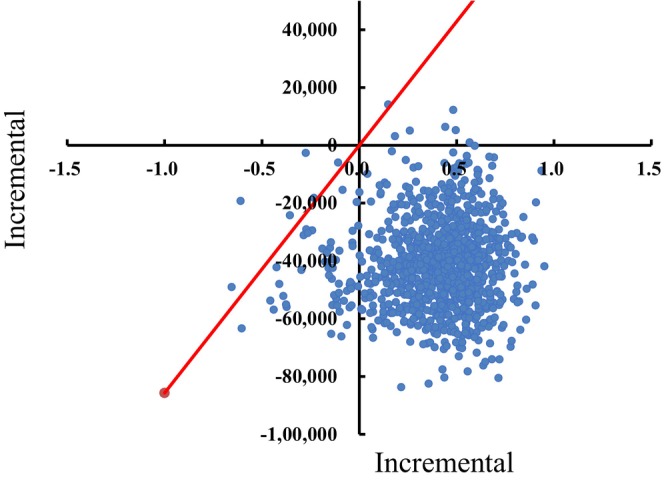
Probabilistic sensitivity analysis: Monte Carlo simulation scatterplot with a threshold of one time the national GDP per capita for TILs testing group versus no TILs testing group. Costs are expressed in Chinese yuan, year 2022values.

**FIGURE 4:**
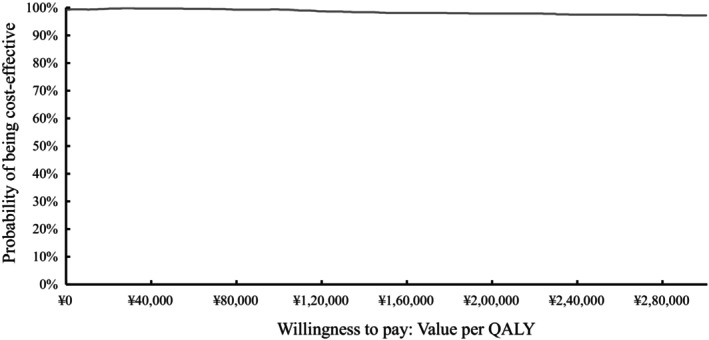
Acceptability curve. The curve demonstrates the probability of the TILs testing group being cost effective compared with the no TILs testing group at a given willingness to pay. ¥: yuan, QALY, quality‐adjusted life year.

The results of the one‐way sensitivity analysis are presented in a tornado diagram (Figure 2). The parameters that significantly affected ICER estimates included utility parameters in the PFS for patients spared chemotherapy and for patients who received chemotherapy, the annual discount rate, and treatment cost after relapse. The cost‐effectiveness plane and cost‐efficiency acceptability curves are presented in Figures 3 and 4, respectively. At a willingness‐to‐pay (WTP) threshold of 85,700 yuan (per‐capita GDP) per QALY, the probability of the cost‐effectiveness of TILs testing for early‐stage TNBC patients was almost 100%.

## 4 DISCUSSION

In this study, we provide economic evidence for the use of TILs testing in early‐stage TNBC patients to determine eligibility for chemotherapy. This has not been reported in other countries to date.

The current standard of treatment for almost all patients with early‐stage TNBC in China and worldwide is cytotoxic chemotherapy. This can lead to overtreatment, which causes patients considerable adverse exposure risks and costs. According to reports from China, the mean out‐of‐pocket costs per breast cancer patient accounts for 55.2% of the average household's non‐food expenditures in China. Notably, health insurance reimbursement rates were relatively low for breast cancer.^40^ Conversely, chemotherapy de‐escalation simultaneously prioritizes patients' quality of life and reduces the pressure on health care systems by avoiding high‐cost treatments that offer no additional or very limited benefit to patients' survival. Our results showed across a range of plausible parameter estimates that the use of TILs testing to identify early TNBC patients with good prognosis is highly cost‐effective compared with the conventional treatment. Considering the ICER value of our results is relatively low (ICERs are less than the per‐capita GDP^41^), there may be implications for decision‐making regarding the use of TILs for de‐escalation of chemotherapy, both in developed countries, and in other developing countries. Sensitivity analysis showed that the cost‐effectiveness results were robust under the uncertainty of the model.

Our analysis had several limitations. First, our results were based on data from published papers and not from prospective studies. Specifically, we used the GetData Graph Digitizer software^31^ to read the coordinate points of PFS and OS curves in relevant published literature for subsequent reconstruction of individual data and estimation of transition probabilities. These reconstructions need to be formally validated within the rigorous framework of prospective studies. The estimates of the utility parameters were derived from literature published in other countries. In general, utility reflects the social and cultural context of a country and may differ across countries. However, there are no available utility parameters generated in China, and calculating these parameters was outside the scope of this study. Second, we relied on the test results of Loi et al.^19^, ^20^ and their definition of clinical outcomes for patients with TNBC. They defined a 30% TILs cutoff based on the top quartile of a large dataset of 2148 patients with TNBC. The TILs Working Group reported that a re‐analysis of three‐ring studies of TILs showed good agreement among pathologists when this cutoff was used. Loi et al.^19^, ^20^ also reported that approximately one‐third of patients had at least a 30% TILs value. Our published review^18^ suggests approximately 42% of patients had a TIL level of exceeding 20%. The sensitivity and specificity were higher for the 20% threshold than for other thresholds. A 20% threshold of TILs may therefore also have better predictive and prognostic effects than a 30% threshold. This is similar to the results of another Chinese study.^42^ Hence, we performed a sensitivity analysis of the 20% threshold and the associated proportion of patients, and we found that the results (ICER = −68426.59) were similar to the results for the 30% threshold (ICER = −86122.87). We therefore conclude that implementing TILs testing to guide de‐escalation of chemotherapy in early TNBC is highly cost‐efficient. Moreover, estimating ICER at different TILs thresholds and identifying the clinical outcomes and economic evidence associated with these cutoffs, remains an important area for future research. Third, our model did not take into account the possibility of local recurrence or minor or long‐term adverse events resulting from chemotherapy. However, this area of research has been explored by most cost‐effectiveness studies of breast cancer. Fourth, although we built a relatively comprehensive model, our simulation is still a simplification of reality. Fifth, substantial uncertainty surrounds the cost‐effectiveness of TILs and additional research to clarify critical assumptions would be prudent. Sixth, it is assumed that all patients with TILs values greater than 30% are spared from chemotherapy, which is an idealized situation. Practically, the clinical decision to administer chemotherapy is complex and requires consideration of patient preferences and other clinical features. However, we cannot identify and simulate all the uncertain factors. This is also a challenge and limitation of economic evaluation in general.

## 5 CONCLUSIONS

Our study demonstrates that, compared with administering chemotherapy to all patients, using TILs as a biomarker to guide chemotherapy de‐escalation in patients with early TNBC was highly cost‐effective from a Chinese health care system perspective. These results might help clinicians in making better decisions about patient risks and benefits before administering cytotoxic chemotherapy to treat patients with early‐stage TNBC. Furthermore, our findings provide a basis for decision‐makers to consider the inclusion of the TILs assay in China's social health insurance benefit package.

## AUTHOR CONTRIBUTIONS

**Shiqi Li:** Data curation (lead); formal analysis (lead); methodology (lead); software (supporting); writing – original draft (lead); writing – review and editing (lead). **Yuhan Liu:** Formal analysis (supporting); methodology (lead); software (lead); writing – review and editing (supporting). **Peigen Zhang:** Data curation (supporting); formal analysis (supporting); resources (lead); writing – review and editing (supporting). **Mengmeng Wang:** Data curation (supporting); resources (supporting). **Lihua Sun:** Supervision (lead); writing – review and editing (supporting).

## FUNDING INFORMATION

This research did not receive any specific grant from funding agencies in the public, commercial, or not‐for‐profit sectors.

## CONFLICT OF INTEREST STATEMENT

The authors declare that they have no competing interests.

## Data Availability

The datasets generated during and/or analyzed during the current study are available from the corresponding author on reasonable request.
